# Complex kinetics and residual structure in the thermal unfolding of yeast triosephosphate isomerase

**DOI:** 10.1186/s12858-015-0049-2

**Published:** 2015-09-03

**Authors:** Ariana Labastida-Polito, Georgina Garza-Ramos, Menandro Camarillo-Cadena, Rafael A. Zubillaga, Andrés Hernández-Arana

**Affiliations:** Área de Biofisicoquímica, Departamento de Química, Universidad Autónoma Metropolitana-Iztapalapa, San Rafael Atlixco 186, Iztapalapa, D.F. 09340 Mexico; Departamento de Bioquímica, Facultad de Medicina, Universidad Nacional Autónoma de México, Coyoacán, D.F. 04510 Mexico

## Abstract

**Background:**

*Saccharomyces cerevisiae* triosephosphate isomerase (yTIM) is a dimeric protein that shows noncoincident unfolding and refolding transitions (hysteresis) in temperature scans, a phenomenon indicative of the slow forward and backward reactions of the native-unfolded process. Thermal unfolding scans suggest that no stable intermediates appear in the unfolding of yTIM. However, reported evidence points to the presence of residual structure in the denatured monomer at high temperature.

**Results:**

Thermally denatured yTIM showed a clear trend towards the formation of aggregation-prone, β-strand-like residual structure when pH decreased from 8.0 to 6.0, even though thermal unfolding profiles retained a simple monophasic appearance regardless of pH. However, kinetic studies performed over a relatively wide temperature range revealed a complex unfolding mechanism comprising up to three observable phases, with largely different time constants, each accompanied by changes in secondary structure. Besides, a simple sequential mechanism is unlikely to explain the observed variation of amplitudes and rate constants with temperature. This kinetic complexity is, however, not linked to the appearance of residual structure. Furthermore, the rate constant for the main unfolding phase shows small, rather unvarying values in the pH region where denatured yTIM gradually acquires a β-strand-like conformation. It appears, therefore, that the residual structure has no influence on the kinetic stability of the native protein. However, the presence of residual structure is clearly associated with increased irreversibility.

**Conclusions:**

The slow temperature-induced unfolding of yeast TIM shows three kinetic phases. Rather than a simple sequential pathway, a complex mechanism involving off-pathway intermediates or even parallel pathways may be operating. β-strand-type residual structure, which appears below pH 8.0, is likely to be associated with increased irreversible aggregation of the unfolded protein. However, this denatured form apparently accelerates the refolding process.

**Electronic supplementary material:**

The online version of this article (doi:10.1186/s12858-015-0049-2) contains supplementary material, which is available to authorized users.

## Background

It is now accepted that many proteins fold and unfold following complex kinetic models [1]. The most detailed kinetic studies of conformational change have been performed on small monomeric proteins by means of rapid mixing or fast temperature jumps, because protein molecules of this sort usually unfold reversibly but with relaxation times ranging from less than a millisecond to a few seconds [2–4]. Previous studies have demonstrated the presence of transiently populated intermediates, apart from the native and unfolded end-states [1, 3]. Intermediate states may be found either on- or off-pathway, and their interconnections may even result in the consolidation of parallel, competing folding-unfolding pathways [5, 6]. Furthermore, the combination of experimental studies and molecular dynamics simulations has provided detailed structural descriptions of the multiple intermediates and transition states involved [7]. Recently, strong emphasis has been placed on the structural characterization of unfolded states, because the presence of residual, native-like structure in parts of an otherwise unfolded polypeptide chain may be implicated in the speed of folding, as well as in the formation of misfolded molecules [8, 9].

However, there are examples of proteins that show very slow unfolding-refolding kinetics in the transition region (i.e., under conditions where the native and unfolded states are both significantly populated at equilibrium). Specifically, when unfolding is promoted by adding GuHCl or urea, slow-unfolding proteins take days to weeks to equilibrate, whereas for fast-unfolding proteins under similar conditions, equilibrium is reestablished in just a few hours [10–12]. Thus, if incubation times in the denaturing agent are not long enough, a slow-unfolding protein would display noncoincident unfolding and refolding profiles as the concentration of denaturing agent is varied (hysteresis). Likewise, hysteresis has been nicely demonstrated in the temperature-induced transitions of at least four proteins: an immunoglobulin light chain (monomer) [13], the Lpp-56 three-stranded α-helical coiled coil [14], and two dimeric triosephosphate isomerases [15, 16]. In these cases, thermal transitions detected by circular dichroism (CD) appear to be consistent with a two-state model with no intermediates.

Regarding trisosephosphate isomerase (TIM), many mesophilic members belonging to this enzyme family have been found to unfold slowly in chemical-denaturation studies, with one or more equilibrium or kinetic intermediates [11, 17, 18]. In contrast, thermal unfolding transitions of TIMs (in the absence of chemical denaturants) usually manifest themselves as monophasic profiles (i.e., simple sigmoidal curves with no evidence of intermediates), as recorded by CD [15, 19–21]. Unfortunately, irreversibility appears as a common feature in thermal unfolding, which has precluded the study of TIM refolding in cooling scans. Nevertheless, Benítez-Cardoza et al. [15] demonstrated that yeast TIM (yTIM) thermal unfolding is highly reversible at low protein concentration (≈0.20 μM), although the unfolding-refolding cycle displays marked hysteresis when a heating-cooling rate of 2.0 °C min^−1^ is used. Attempts to achieve near-equilibrium transition profiles by decreasing the scan rate led to pronounced irreversibility [15].

At a fixed temperature, kinetic data for yTIM unfolding registered by far-UV circular dichroism (CD) in a restricted time span, are well-fitted by single exponential curves, whereas near-UV CD and fluorescence indicate biphasic kinetics. Refolding data are consistent with a second-order reaction [15]. Unlike yeast TIM, the enzyme from *Trypanosoma cruzi* (TcTIM) shows completely irreversible, temperature-induced denaturation, even at low protein concentration. Kinetic studies of this protein found that denaturation is a complex process in which two or three phases are clearly seen [19]. A common finding for both yTIM and TcTIM is that their denatured states appear to conserve some kind of residual structure based on calorimetric data [15, 19].

This work mainly focuses on determining the kinetic characteristics of temperature-induced yTIM unfolding in aqueous solution over long durations and in a wide pH range. Regardless of pH, three kinetic phases were observed, although the small-amplitude faster phase was detected at only low temperatures. The relative amplitudes of the second and third phases vary with temperature in a way that seems difficult to explain by a sequential mechanism. The results thus evidence that the kinetics of yTIM thermal unfolding is more complex than previously thought. Furthermore, residual secondary structure was found in denatured yTIM below pH 8.0. Because this residual structure appears to be associated with the loss of refolding ability, its presence may indicate that misfolded, aggregation-prone structures are formed at high temperature. Molecular dynamics simulations showed that yTIM has a tendency to suffer α-to-β transitions when unfolded at high temperature, but this method does not properly reproduce the marked effect of pH on the structure of the thermally unfolded protein.

### Materials

Overexpresion and purification of wild-type *Saccharomyces cerevisiae* TIM (yTIM) was carried out as described elsewhere [22]. Mass spectrometry (Additional file 1) and SDS-PAGE showed that the obtained enzyme was homogeneous. Enzymatic activity was determined by the coupled assay with α-glycerophosphate dehydrogenase (α-GDH), using D-glyceraldehyde 3-phosphate (DGAP) as the TIM substrate [23]. Assays were performed at 25.0 °C in 1.0 mL of 0.1 M triethanolamine buffer (pH 7.4) containing 10 mM EDTA, 0.20 mM NADH, 0.02 of α-GDH, and 2.0 mM DGAP; the reaction was started by the addition of 3.0 ng of yTIM, and NADH oxidation was followed by the change in absorbance at 340 nm. The catalytic efficiency (*k*_cat_/*K*_M_) of this enzyme was 5.0 x 10^6^ s^–1^ M^–1^, a value similar to that reported previously [24, 25].

### Circular dichroism spectra

Circular dichroism (CD) spectra were obtained with a JASCO J-715 instrument (Jasco Inc., Easton, MD) equipped with a Peltier-type cell holder for temperature control and stirring with a magnetic bar. Cells of 1.00-cm path length were used to keep the protein concentration near 10 μg mL^−1^ (0.19 μM). Although this somewhat restricted the lower wavelength limit of data registering, a low concentration is mandatory to observe reversible thermal unfolding scans [15]. CD spectral data are reported as mean residue ellipticity, [θ], which was calculated as [θ] = 100 θ/(*C l*); in this expression θ is the measured ellipticity in degrees, *C* is the mean residue molar concentration (mean residue *M*_r_ = 107.5), and *l* is the cell path length in centimeters.

### Thermal transitions

Conformational changes induced by heating or cooling of yTIM were continuously monitored by following the ellipticity at 220 nm while temperature was varied at 2.0 °C min^−1^. Samples (≈0.19 μM) were placed in a 1.00-cm cell with a magnetic stirrer, and the temperature within the cell was registered by the external probe of the Peltier-type accessory. Refolding profiles were registered immediately after the unfolding transitions had been completed.

### Kinetics studies

Unfolding kinetics tracings were registered by following ellipticity changes at 220 nm, as described previously [15, 25]. Unfolding was initiated by adding a small aliquot of concentrated TIM solution to a 1.00-cm cell containing buffer equilibrated at the temperature selected for each experiment. Within the cell, the temperature reached ±0.15 °C of the final equilibrium value in about 15 s. The final protein concentration was 0.19 μM in most cases. Essentially, the same procedure was used for monitoring changes in intrinsic fluorescence over time. In this case, experiments were carried out in a K2 spectrofluorometer (ISS, Champaign, IL), which had a Peltier accessory. Protein samples were excited at 292 nm, and the light emitted at 318 nm was collected. Kinetic data were analyzed using a triple exponential decay equation:

1 $$ y={y}_0+{A}_1\left[ \exp \left(-{\lambda}_1t\right)-1\right]+{A}_2\left[ \exp \left(-{\lambda}_2t\right)-1\right]+{A}_3\left[ \exp \left(-{\lambda}_3t\right)-1\right] $$

where *y* is the physical observable monitored as a function of time *t*, and *y*_0_ is the initial value of the observable. *A*_*i*_ and λ_*i*_ represent the observed amplitude and rate constant, respectively, for the *i*th exponential phase. In some cases, only two exponential terms were required for satisfactory curve fitting.

In refolding experiments, yTIM samples were first subjected to unfolding for 10 min at 63.0 °C. Then, the temperature control of the CD spectrometer Peltier accessory was set to a value 4.0 °C below the temperature intended for the study of the refolding reaction (42.0 °C) to allow for fast cooling of the sample (≈15 °C min^−1^). The final temperature value (42.0 °C) was entered into the cell-holder control when the solution in the cell was 0.5 °C above that value, and the CD signal was registered thereafter. Inside the cell, temperature came to equilibrium (±0.15 °C) in approximately 40 s.

### Molecular dynamics simulations

Molecular dynamics (MD) simulations were performed using GROMACS 4.5.4 software [26] with the GROMOS96 53A6 force-field [27]. The side-chain ionization states in the protein at the pH values simulated (6.7, 7.4, and 8.0) were established using *pK*_*a*_ values estimated with PROPKA [28]. Dimeric yTIM (PDB ID: 1YPI) was placed in the center of a periodic dodecahedral box with 10 Å between the protein and the edge of the box. To simulate the solvent conditions at pH 6.7 (7.4; 8.0), a total of 21,763(21,757; 21 751) SPC water molecules, 12 (16; 20) sodium ions, and 7 (10; 12) chloride ions were needed to fill the box, neutralize the net protein charge, and reach the experimental ionic strength of 0.015 M (0.022 M; 0.027 M).

Prior to MD simulations, the system was relaxed by energy minimization, followed by 100 ps of thermal equilibration under the position restraints of protein heavy atoms through a harmonic force constant of 1000 kJ mol^−1^ nm^−1^. MD simulation was performed using an NPT ensemble at 423 K and 1.0 bar for 100 ns. A LINCS algorithm was applied to constrain the length of all covalent bonds [29], and a 2-fs time step was used. A cutoff of 1.0 nm was applied for short-range electrostatic and van der Waals interactions, while the long-range electrostatic forces were treated using the particle mesh Ewald method [30]. Two replicas were simulated at each solvent condition.

## Results and discussion

### Unfolding-refolding thermal transitions

**Fig. 1:**
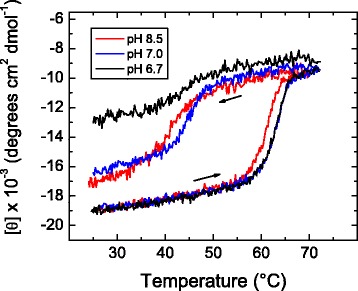
Thermal unfolding-refolding transitions of yTIM at selected pH values. The ellipticity at 220 nm was monitored while samples were heated or cooled at 2.0 °C min^−1^. *Arrows* indicate whether the temperature increased or decreased in scans

Denaturation (unfolding) and renaturation (refolding) of yTIM were followed by continuous monitoring of the ellipticity (220 nm) under a constant heating or cooling rate of 2.0 °C min^−1^. Temperature scanning profiles recorded at three different pH values are shown in Fig. 1. These profiles display the hysteresis phenomenon previously observed for yTIM [15, 16, 25], which indicates that unfolding and refolding events occur under kinetic control at the imposed scanning rate [14, 15]. It is clear that pH has an influence on the kinetic stability of the protein, because the apparent *melting temperature* is displaced to lower values at pH 8.5. Despite this pH effect, all the unfolding traces appear as sigmoid curves, with no evidence of stable intermediates. However, the total ellipticity change at pH 6.7 that takes place upon denaturation seems slightly larger (Fig. 1). It must be noted that the up-temperature scans in Fig. 1 were not allowed to proceed to higher temperatures to avoid reactions that make the process irreversible and thus decrease the extent of refolding on down-temperature scans [15].

**Fig. 2:**
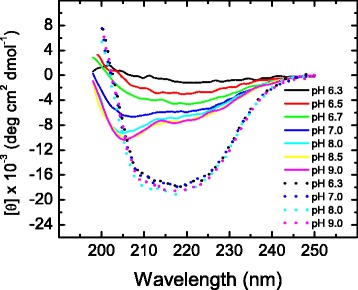
Far-UV CD spectra of thermally unfolded yTIM at different pH values. Protein samples were allowed to unfold by continuous heating (2 °C min^−1^) until the end of the transition (cf. Fig. 1) and then left to stand at 70.0 °C for 10 min before recording their spectra. For comparison, spectra of native yTIM at various pH values are also shown (*dotted lines*)

In a different set of experiments, denatured samples of the enzyme were left to stand at 70 °C for 10 min to ensure that unfolding had been completed before their CD spectra were recorded. Spectra shown in Fig. 2 indicate that the native α/β secondary structure of yTIM is rather insensitive to pH and is largely lost upon heating at all pH values, as judged by the decrease in magnitude in the 208–222 nm region at high temperature (Fig. 2). However, the spectrum of heat-denatured yTIM shows striking changes as pH is varied. Above pH 8.0, the spectral shape and signal magnitude of the denatured enzyme are typical of small and medium-size proteins (e.g., hen-egg lysozyme, ribonuclease A, cytochrome C, staphylococcal nuclease, cysteine proteinases) when unfolded at high temperatures in the absence of denaturant agents (see, for example, CD spectra of native and thermally unfolded lysozyme in Additional file 2). This spectral type is characterized by a negative band of approximately 10 × 10^3^ deg cm^2^ dmol^−1^ centered at 202–204 nm, along with a broad negative shoulder with magnitude around 5 × 10^3^ deg cm^2^ dmol^−1^at longer wavelength [31, 32]. Below pH 8.0, the spectra of heat-denatured yTIM progressively decrease in magnitude and acquire a shape typical of all-β proteins [33], thus pointing to the presence of *residual* secondary structure in the denatured enzyme.

Regarding yTIM refolding in cooling-down scans, it is evident that this process becomes increasingly irreversible as the pH decreases below pH 7.0, as judged from the extent of recovery of native yTIM ellipticity shown in Fig. 1. To gain detailed information on the influence of pH in the unfolding and refolding events, further kinetic experiments were carried out.

### Unfolding kinetics

**Fig. 3:**
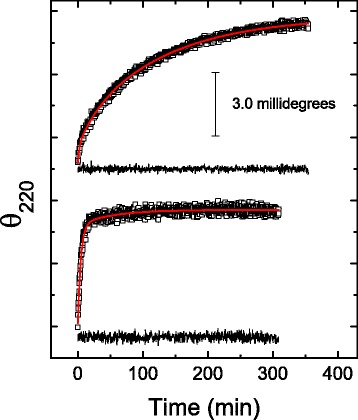
Kinetics of yTIM unfolding at pH 8.0, as followed by far-UV CD at 220 nm. Data shown correspond to 54.5 °C (*upper tracing*) and 60.0 °C (*lower tracing*). *Red lines* are least-squares fits of triple (*upper curve*) or double (*lower curve*) exponential decay equations to experimental data (see Methods). Residuals from fit (*black lines*) are shown below each kinetic tracing

Kinetic studies were carried out by monitoring the time course of ellipticity at 220 nm. Experiments examining a large temperature interval were done at pH 8.0 and 6.7, where the CD spectra of denatured yTIM showed distinct features. The results at pH 8.0 indicate that at relatively high temperatures (60.0 °C and above), the loss of secondary structure shows double exponential behavior (Fig. 3), with phases well separated on the time scale. Indeed, over a restricted time interval, a single exponential-decay equation can fit the experimental data reasonably well. Only when data were recorded over a long time did a second phase become readily apparent, but this phase had small amplitude. Nevertheless, at low temperature, triple exponential behavior was observed (Fig. 3). The fastest phase, which conveys a minor ellipticity change, occurred too fast for accurate assessment of the kinetic constant by the manual-mixing method (i.e., time constant of about 20 to 100 s). This fast phase seems to be completely lost within the dead time in experiments at high temperature. Hereafter, the observed rate constants are referred to as λ_1_, λ_2_, and λ_3_, in descending order of their magnitudes. Unfolding of yTIM at pH 6.7 showed similar behavior, with two and three kinetic phases at high and low temperature, respectively, as shown in Additional file 3.

CD spectra were recorded near the end of the unfolding process when the slowest phase was more than 98 % complete (these experiments required recordings of kinetic data for more than nine hours in the case of low temperatures). The *final* spectra appeared nearly identical, notwithstanding the temperature at which the kinetics was studied (see Additional file 4 for results obtained at pH 8.0). Furthermore, at a given pH, the spectral shape and magnitude observed at the end of unfolding were both similar to those illustrated in Fig. 2. In other words, the final conformation achieved by the protein seems to be independent of the temperature (in the range studied), but is otherwise strongly affected by pH.

The voltage applied to the phototube of the CD instrument, which is proportional to the absorbance, was simultaneously recorded. The measurements indicated that changes in ellipticity associated with the first two phases are accompanied by only small changes (5.0 % or less) in the absorbance of the protein solution (see Additional file 5). Such small changes are known to occur due to alterations in the secondary and, to less extent, the tertiary structure of proteins and polypeptides [34]. However, a relatively large absorbance increment (approximately 10.0 % of the protein absorbance) was linked to the slower CD-detected kinetic phase. It is likely that this apparent increment comes from the scattering of light by aggregates of unfolded protein molecules.

**Fig. 4:**
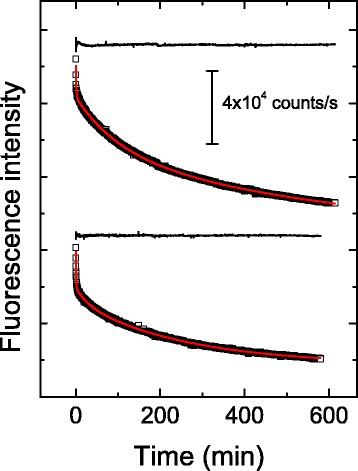
Kinetics of yTIM unfolding at pH 8.0, as followed by fluorescence intensity. Data shown correspond to 55.0 °C (*upper tracing*) and 60.0 °C (*lower tracing*). Protein samples were excited at 292 nm, and the light emitted at 318 nm was collected. Red lines are least-squares fits of triple exponential decay equations to experimental data (see Methods). Residuals from fit (*black lines*) are shown above each kinetic tracing

Monitoring of the denaturation kinetics by changes in the fluorescence intensity also showed that this is a complex process (Fig. 4) in which there is a progressive decrease of intensity (at the wavelength of maximum emission by native yTIM). Overall, comparison of the plots shown in Figs. 3 and 4 indicates that progressive loss of secondary structure upon denaturation is accompanied by a quenching of the fluorescence signal of tryptophan residues, which in turn likely reflects either the exposure of these residues to the aqueous solvent or less constraint by the environment [35]. Notwithstanding the temperature, three exponential terms were required to fit fluorescence data. As in CD experiments, the first fluorescence-detected phase was too fast (time constant of about 25 s) for an accurate determination of its rate constant. At low temperature (55.0 °C), the rate constant for the second phase had a value similar to that of λ_2_ from CD experiments (the two values differed by 50–80 %). At 62.0 to 64.0 °C, however, it was the first fluorescence-detected rate constant that was consistent with λ_2_. Furthermore, the decrease in the fluorescence intensity extended over a much longer time than the change in ellipticity (i.e., the rate constant for the slowest phase was approximately three- to fourfold smaller when determined from fluorescence than from CD). These markedly different values suggest that the slowest phase comprises several elementary steps that respond differently to the spectroscopic probes employed. For instance, formation of molecular aggregates can conceivably occur with little or no change in secondary conformation, but with an otherwise significant fluorescence quenching of tryptophan residues.

### Kinetic model for yTIM unfolding

**Scheme 1:**
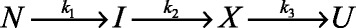
Kinetic model for three sequential first-order reactions

**Scheme 2:**
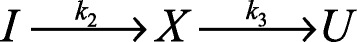
Simplified kinetic model involving only two first-order steps

The simplest model accounting for the results obtained from CD would be that of three sequential reactions (Scheme 1), with native and unfolded yTIM (N and U, respectively) and two intermediate species (I and X):

In this model, each of the three λ values determined from data analysis (eqn. 1) is identical to each one of the microscopic rate constants *k*_1_, *k*_2_, and *k*_3_. As mentioned, neither the rate constant nor the amplitude of the faster phase could be accurately determined from experiments at the lowest temperatures studied. Moreover, this phase was apparently lost within the dead time of experiments performed at high temperature. Fortunately, because *k*_1_ seems to be 15–20 times larger than *k*_2_, the first kinetic step occurs on a much shorter time scale than the other steps and can be regarded as kinetically separated from the other events, at least in a first approximation. This implies that amplitudes *A*_2_ and *A*_3_ reflect changes involved solely with steps I → X → U. Therefore, the kinetic model can be simplified to a two-step model (Scheme 2).

Equations describing the evolution in time of the fraction of each species are well known [36, 37]. By denoting the characteristic ellipticity of each species as θ_I_, θ_X_, and θ_U_, it can be shown that (see Additional file 6):

2 $$ \left({\uptheta}_{\mathrm{X}}-{\uptheta}_{\mathrm{I}}\right)/\left({\uptheta}_{\mathrm{U}}-{\uptheta}_{\mathrm{I}}\right)={k}_3/{k}_2-\left[\left({k}_3-{k}_2\right)/{k}_2\right]\left[{A}_2/\left({A}_2+{A}_3\right)\right] $$

and

3 $$ \left({\uptheta}_{\mathrm{U}}-{\uptheta}_{\mathrm{X}}\right)/\left({\uptheta}_{\mathrm{U}}-{\uptheta}_{\mathrm{I}}\right)=-\left[\left({k}_3-{k}_2\right)/{k}_2\right]\left[{A}_3/\left({A}_2+{A}_3\right)\right] $$

**Fig. 5:**
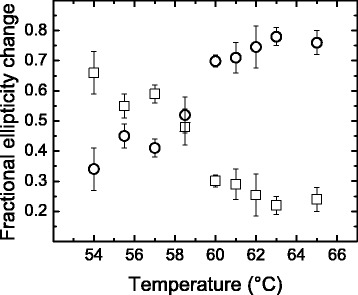
Fractional structural change for the two kinetic steps in Scheme 2 at different temperatures. Fractional changes in ellipticity were calculated from eqns. 2 and 3 from values of the rate constants and amplitudes determined at pH 6.7. Data for step I → X, i.e., (θ_X_−θ_I_)/(θ_U_−θ_I_), are shown as circles; data for step X → U, i.e., (θ_U_−θ_X_)/(θ_U_−θ_I_), are shown as *squares*

The two equations above were used to compute (θ_X_−θ_I_)/(θ_U_−θ_I_) and (θ_U_−θ_X_)/(θ_U_−θ_I_), which give the ellipticity change as a fraction of the total change for each step in Scheme 2. The results indicate that the degree of unfolding occurring during the I → X step (normalized to a total unitary change) would vary from 0.35 to 0.70 over a temperature range of 11 °C (Fig. 5). For the X → U step, a concomitant decrease in the degree of unfolding would take place. Admittedly, it seems unlikely that the conformation of intermediate species would vary so drastically within such a narrow temperature range. Alternatively, these results may point to the presence of an off-pathway intermediate or even different, parallel unfolding pathways with predominance that changes with temperature.

### Temperature dependence of unfolding rate constants

**Fig. 6:**
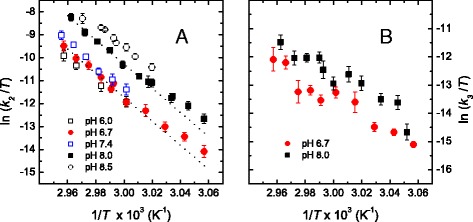
Eyring plots for the rate constants *k*_2_(**a**) and *k*_3_(**b**) at selected pH values. Rate constants were determined from far-UV CD kinetic experiments. Dotted lines in (**a**) are linear fits performed with data corresponding to temperatures above 60.0 °C for pH 6.7 and 8.0

Further studies on the denaturation of the enzyme were also performed at other pH values but over a restricted temperature range to determine the *activation* parameters that control the temperature dependence of *k*_2_ and *k*_3_. Results for selected pH values are shown in Fig. 6 as Eyring plots, which agree with the well-known equation:

4 $$ \ln \left(k/T\right)= \ln E+\varDelta {S}^{\ddagger }/R-\left(\varDelta {H}^{\ddagger }/R\right)\left(1/T\right) $$

where *k* is the rate constant for an elementary reaction, *T* is the absolute temperature, *E* stands for a preexponential factor, and Δ*S*^‡^ and Δ*H*^‡^ represent the activation entropy and enthalpy, respectively. Figure 6a shows that plots corresponding to *k*_2_ follow linear trends when a narrow temperature range is considered. This linearity was observed before for yTIM and has been found for a large number of other proteins [15]. However, in the cases of pH 6.7 and 8.0, at which larger temperature intervals were examined, Eyring plots appear slightly curved upwards in the low temperature region. This might be due to a shift between parallel unfolding pathways with different *activation* enthalpies [38]; that is, unfolding would switch from one predominant pathway to another as the temperature varies, in agreement with the interpretation mentioned for the change with temperature of the computed degree of unfolding for step I → X. However, a nonzero activation heat capacity cannot be ruled out as the origin of the curvature.

From Eyring plots, such as those in Fig. 6a, Δ*H*_2_^‡^ was determined between pH 6.0 and 8.5. It must be noted that values of *k*_2_ were determined from data registered in a temperature region in which the unfolding degree accompanying step I → X remains relatively constant (i.e., from 60 to 65 °C, cf. Fig. 5). Therefore, *k*_2_ can be assigned to a single predominant pathway. Overall, the value of Δ*H*_2_^‡^was about 450 kJ mol^−1^at pH 6.0–8.0 and showed a slight decrease (≈15 %) at pH 8.5 (data not shown). In contrast, Eyring plots for *k*_3_ display a linear but ill-defined trend (Fig. 6b), suggesting that the slowest kinetic phase is indeed composed of several elementary steps. It is also seen that *k*_3_ is much less temperature dependent than *k*_2_.

**Fig. 7:**
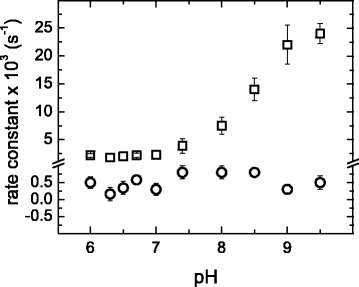
Variation of rate constants *k*_2_ and *k*_3_ with pH. Data for *k*_2_ (squares) and *k*_3_ (circles) were determined from far-UV CD kinetic experiments at 60.0 °C

The effect of pH on *k*_2_ and *k*_3_ was examined over a longer interval of pH values at constant temperature; 60.0 °C was chosen, because of the single apparent pathway at this temperature, and the unfolding process was slow enough to allow for determining the value of *k*_2_over an extended pH range. Results are shown in Fig. 7, which shows that pH-induced changes in *k*_2_ resemble the sigmoid titration curve for an ionizable group with an approximate p*K*_*a*_ of 8.5. Because this value of p*K*_*a*_ is close to that of a thiol group, it may be hypothesized that a cysteine residue is responsible for the behavior observed for *k*_2_. In this regard, it has been proposed that Cys126, which is a residue that is conserved with the family of TIM enzymes, plays an important role in the stability of this protein [24]. In contrast, *k*_3_ values showed no defined variation with pH, again suggesting that the step X → U actually comprises multiple individual reactions.

### Refolding of yTIM

As reported previously [15, 24, 25], the kinetics of yTIM refolding at low protein concentration (0.13–0.75 μM) and in a certain temperature range is slow enough to be monitored without resorting to fast temperature-jump techniques. By using the procedure described in the Methods section, we followed the recovery of secondary structure under two pH conditions. These studies were aimed at determining the effect of the *residual* native-like structure of unfolded yTIM (which is clearly observed at pH 6.7) on the refolding ability of the enzyme. For this purpose, yTIM samples were allowed to unfold (in the cell of the CD instrument) for 10 min at 63.0 °C. These conditions ensured ca. 85 % (pH 6.7) or 99 % (pH 8.0) unfolding, as judged by the ellipticity signal. After that, the protein solution was cooled to 42.0 °C to record the refolding reaction. Additional file 7 shows that at pH 6.7 the enzyme refolds faster than at pH 8.0. In both cases, however, refolding tracings are adequately described by a second-order kinetics equation, as determined previously [15, 24].

**Fig. 8:**
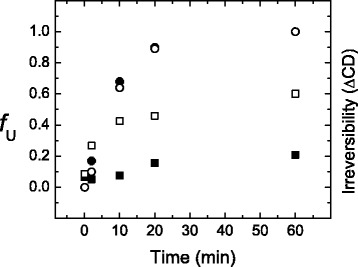
Time course for the appearance of irreversibility on the unfolding of yTIM. Samples of yTIM were unfolded (63.0 °C) for different time spans, and then cooled to 25 °C for recording of CD spectra. *Irreversibility* was then calculated as the difference in ellipticity (220 nm) between cooled-down samples and native yTIM, normalized to the ellipticity of the native protein. Irreversibility data are represented by open (pH 6.7) or solid (pH 8.0) squares. Open (pH 6.7) and solid (pH 8.0) circles correspond to the fraction of unfolded protein, *f*_U_, which was calculated from eqn. S3 in Additional file 5

To explore the effect of the residual structure on the reversibility of the unfolding process, samples of yTIM were unfolded for different time spans and then cooled to 25 °C to record CD spectra. As a quantitative indicator of irreversibility, the difference in ellipticity (220 nm) between cooled-down samples and native yTIM, normalized to the ellipticity of the native protein, was used. The results are shown in Fig. 8, together with the fractional values of U (*f*_U_) in Scheme 2. Experimentally determined values of the kinetic constants *k*_2_ and *k*_3_ were used to calculate the time variation of *f*_U_ according to eqn.S3 in Additional file 6. An inspection of the plots in the figure makes it evident that irreversibility is more intense at pH 6.7 than pH 8.0, as expected from the thermal scan results (cf. Fig. 1). At the lower pH, however, irreversibility begins with early unfolding times and approximately parallels the formation of *f*_U_. In contrast, the onset of irreversibility at pH 8.0 is delayed and thus appears as a late event in unfolding, which takes place after the final U state becomes largely populated. This suggests again that the slowest CD-detected kinetic phase does not represent an elementary step. Reactions that lead to irreversibility probably do not involve major changes in secondary conformation and are therefore silent in CD studies.

In summary, results from refolding studies indicate that yTIM refolds faster from the denatured state with residual structure, although such denatured state decreases the folding efficiency (i.e., the amount of native protein recovered upon refolding). Thus, it may be thought that under physiological conditions (pH near neutrality, 37 °C) the advantage of a fast folding process overcomes the difficulties posed by some degree of irreversibility. Furthermore, because irreversibility appears to be related to the time unfolded (denatured) YTIM stays at moderate to high temperatures [15], the problem of a low folding efficiency may be of less significance for mesophilic organisms such as *Saccharomyces cerevisiae*.

### Residual structure

As mentioned, thermally denatured yTIM retains a high content of β structure below pH 8.0 (see Fig. 2), which is implicated in reactions leading to irreversibility. This type of secondary structure has been found to be refractory to temperature in thermophilic and mesophilic proteins [39, 40], whereas in other instances, such as in apomyoglobin, β structure appears to be formed at elevated temperature [31] as a result of α-to-β transitions [41]. Furthermore, molecular dynamics simulations have shown that certain all-α peptides, and even full-length proteins, may be transformed to all-β structures [42, 43]. We carried out preliminary MD simulations to investigate whether this method can reproduce the structural differences in denatured yTIM that were experimentally observed when pH is varied. Simulations run at 400 K for 100 ns showed that helixes are completely lost after 75 ns, regardless of the pH value. Conversely, β-strands actually seem to be formed during the simulation, but they are slightly more abundant at pH 6.7 than at pH 8.0 (Additional file 8). Although preliminary, these results are encouraging, for they indicate that some regions in the polypeptide sequence of yTIM have a tendency to undergo α-to-β transitions. In contrast, the effect of pH does not appear to have been properly taken into account by the MD method used here, and thus deserves to be studied further.

## Conclusions

Two experimental approaches were used to study the influence of pH on the temperature-induced unfolding of yeast triosephosphate isomerase (yTIM). Temperature-scan experiments showed that unfolding profiles (monitored by CD) appear as monophasic transitions, with no evidence of intermediate species. pH was found to affect the *kinetic stability* of the protein based on shifts in the *melting temperature* (*T*_*m*_). Furthermore, below pH 8.0, CD spectra of heat-denatured yTIM gradually changed in shape to look like those for proteins rich in β-strands, but otherwise, the unfolded protein became prone to aggregate.

**Scheme 3:**
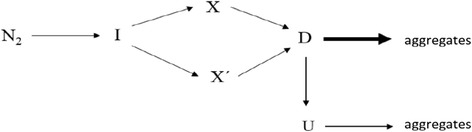
Proposed model for yTIM unfolding

Despite the apparent simplicity of thermal profiles, kinetic studies performed at constant temperature clearly showed the presence of up to three kinetic phases, irrespective of pH (i.e., at high temperatures, the fastest phase was completely lost within the experimental dead time). Because the relative values of the kinetic constants suggested that the fastest phase is indeed *decoupled* from the other two, we analyzed the kinetic constants and amplitudes of the two slowest phases according to a two-step sequential mechanism. Results from the analysis, however, pointed to a more complex actual mechanism, such as one that involves parallel pathways. The temperature dependence of the rate constants appears to lend some evidence to this proposal. A simple model for yTIM unfolding that accounts for the information summarized above is shown in Scheme 3, where N_2_ stands for the native dimer, I and X represent partially unfolded intermediates, and D and U are used to symbolize, respectively, the denatured form with β-strand residual structure and the thermally unfolded state of yTIM.

In summary, it was shown that the temperature-induced denaturation of yTIM reveals itself as a complex process when followed for a long time and over an ample temperature range. Further investigation over a wide pH range showed that the kinetic stability of yTIM responds to the titration of an ionizable group with *pK*_*a*_ ≈ 8.5. Refolding studies, on the other hand, indicated that the refolding ability of the unfolded protein decreases under pH conditions that favor the formation of residual, β-strand-like structures in heat-denatured yTIM, even though refolding is faster under such conditions. Moreover, most of the reactions leading to irreversibility occur late in the unfolding process and are not detected by CD. Finally, as demonstrated in molecular dynamics simulations, yTIM unfolding shows α-to-β transition behavior, albeit with no discrimination of the experimentally observed pH effect.

## Additional files

Additional file 1:
**Mass Spectrum of isolated yTIM.** (PDF 72 kb) Additional file 2:
**Native and thermally unfolded hen-egg lysozyme CD spectra.** (PDF 67 kb) Additional file 3:
**Kinetics of yTIM unfolding at pH 6.7, as followed by CD.** (PDF 210 kb) Additional file 4:
**Far-UV CD spectra of yTIM near the end of the unfolding kinetics process.** (PDF 89 kb) Additional file 5:
**Kinetics of yTIM unfolding detected by light absorption.** (PDF 142 kb) Additional file 6:
**Derivation of eqns.** 2 **and** 3 **in text.** (PDF 113 kb) Additional file 7:
**Refolding kinetics of yTIM as followed by CD.** (PDF 133 kb) Additional file 8:
**Molecular dynamics simulations of yTIM unfolding.** (PDF 113 kb)

## Abbreviations

yTIM: Triosephosphate isomerase from yeast (*Saccharomyces cerevisiae*)
CD: Circular dichroism
MD: Molecular dynamics
