# Supplementary material for: Complex kinetics and residual structure in the thermal unfolding of yeast triosephosphate isomerase
Source: BMC Biochem. 2015 Sep 3;16:20. doi: 10.1186/s12858-015-0049-2 (PMC4558838; doi:10.1186/s12858-015-0049-2)
Supplement: Additional file 1: — Mass Spectrum of isolated yTIM. (PDF 72 kb) [file 12858_2015_49_MOESM1_ESM.pdf]

Additional file 1

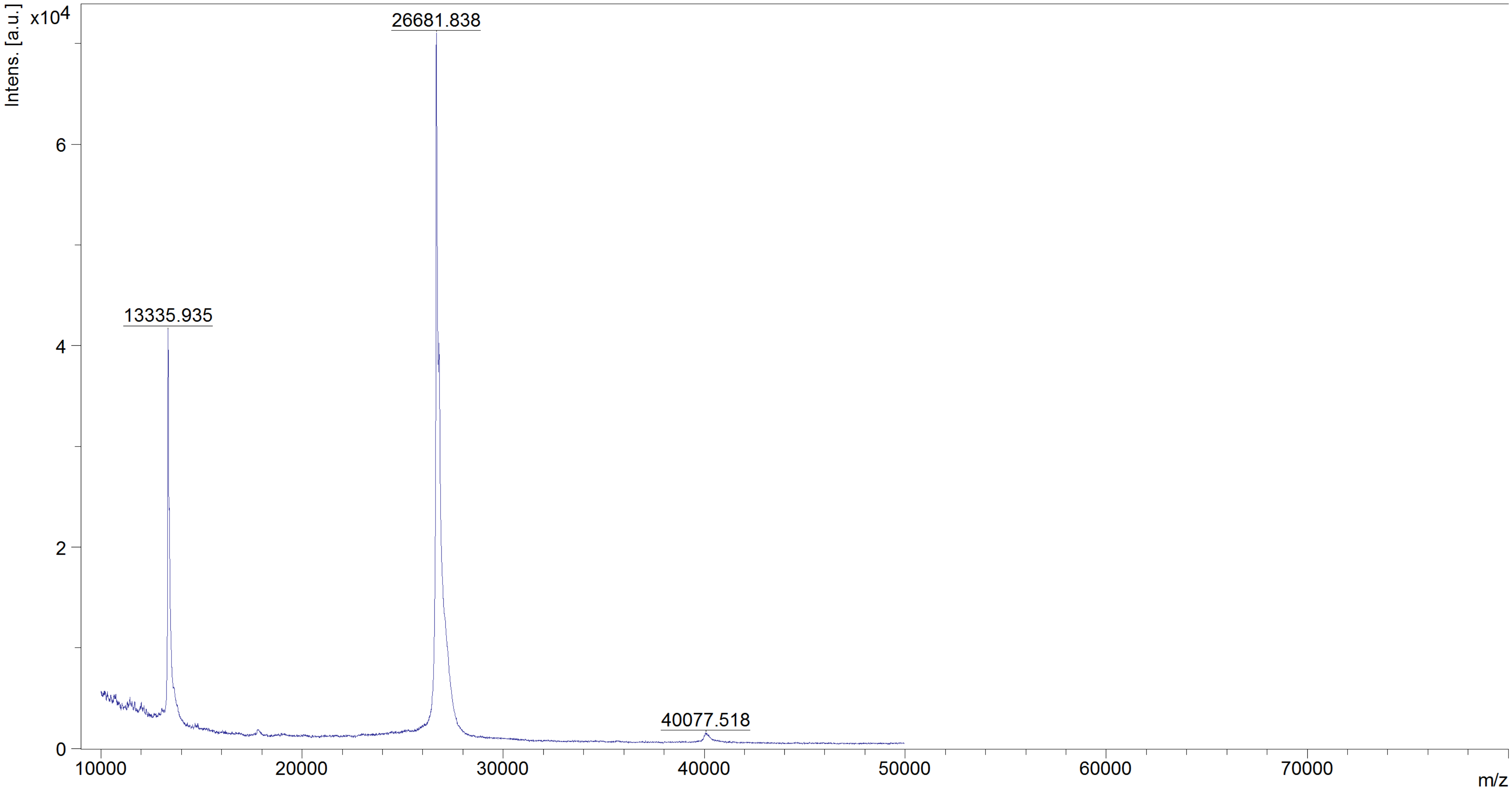

Mass spectrum of yTIM obtained in a MALDI-TOF/TOF AutoFlex Bruker instrument. 2,5 dihydroxybenzoic acid was used as matrix.
