# Supplementary material for: Complex kinetics and residual structure in the thermal unfolding of yeast triosephosphate isomerase
Source: BMC Biochem. 2015 Sep 3;16:20. doi: 10.1186/s12858-015-0049-2 (PMC4558838; doi:10.1186/s12858-015-0049-2)
Supplement: Additional file 2: — Native and thermally unfolded hen-egg lysozyme CD spectra. (PDF 67 kb) [file 12858_2015_49_MOESM2_ESM.pdf]

Additional file 2

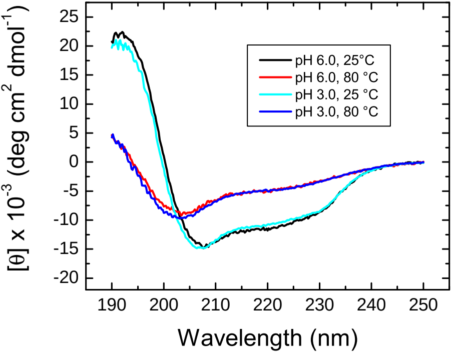

Far-UV CD Spectra of native and thermally unfolded hen-egg lysozyme in aqueous solution
