# Supplementary material for: Complex kinetics and residual structure in the thermal unfolding of yeast triosephosphate isomerase
Source: BMC Biochem. 2015 Sep 3;16:20. doi: 10.1186/s12858-015-0049-2 (PMC4558838; doi:10.1186/s12858-015-0049-2)
Supplement: Additional file 3: — Kinetics of yTIM unfolding at pH 6.7, as followed by CD. (PDF 210 kb) [file 12858_2015_49_MOESM3_ESM.pdf]

Additional file 3

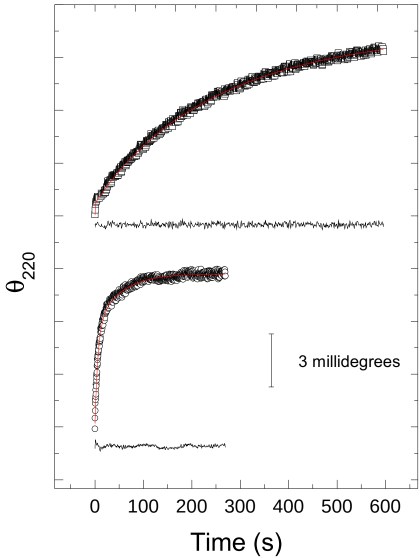

Kinetics of yTIM unfolding at pH 6.7, as followed by far UV-CD. Data shown correspond to 54.0°C (upper trace) and 60.0°C (lower trace). Red lines are least-squares fits of triple (upper curve) or double exponential decay equations to experimental data. Residuals from fit (black lines) are shown below each kinetic trace.
