# Supplementary material for: Complex kinetics and residual structure in the thermal unfolding of yeast triosephosphate isomerase
Source: BMC Biochem. 2015 Sep 3;16:20. doi: 10.1186/s12858-015-0049-2 (PMC4558838; doi:10.1186/s12858-015-0049-2)
Supplement: Additional file 4: — Far-UV CD spectra of yTIM near the end of the unfolding kinetics process. (PDF 89 kb) [file 12858_2015_49_MOESM4_ESM.pdf]

Additional file 4

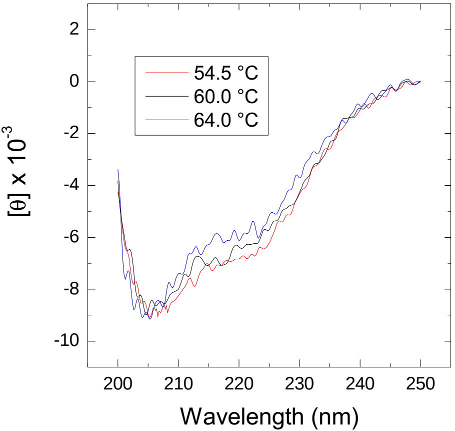

Far UV-CD spectra of yTIM near the end of the unfolding kinetics process. Unfolding was allowed to proceed to 98% or more, at the three different temperatures (pH 8.0), before spectra were registered.
