# Supplementary material for: Complex kinetics and residual structure in the thermal unfolding of yeast triosephosphate isomerase
Source: BMC Biochem. 2015 Sep 3;16:20. doi: 10.1186/s12858-015-0049-2 (PMC4558838; doi:10.1186/s12858-015-0049-2)
Supplement: Additional file 5: — Kinetics of yTIM unfolding detected by light absorption. (PDF 142 kb) [file 12858_2015_49_MOESM5_ESM.pdf]

# Additional file 5

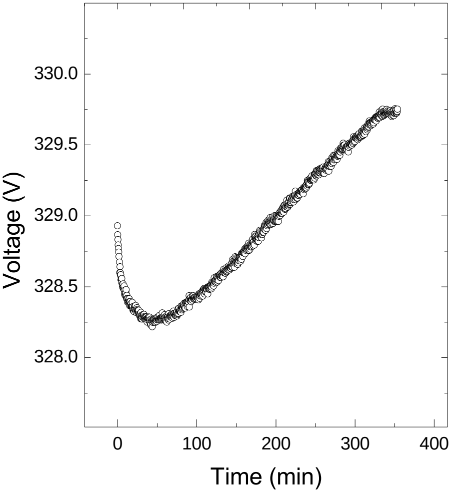


Kinetics of yTIM unfoldig as detected by light absorption (220 nm). Data shown correspond to an experiment, carried out at 54.5 °C and pH 8.0, in which the voltage applied to phototube of the CD instrument was monitored as a function of time.
