# Supplementary material for: Complex kinetics and residual structure in the thermal unfolding of yeast triosephosphate isomerase
Source: BMC Biochem. 2015 Sep 3;16:20. doi: 10.1186/s12858-015-0049-2 (PMC4558838; doi:10.1186/s12858-015-0049-2)
Supplement: Additional file 6: — Derivation of eqns. 2 and 3 in text. (PDF 113 kb) [file 12858_2015_49_MOESM6_ESM.pdf]

Additional file 6

Derivation of equations (2) and (3) in text

For a kinetic model with two consecutive irreversible steps, expressions giving the time course of changes in the concentration of the molecular species involved can be found elsewhere [36,37]. Those equations may be easily modified to obtain the time dependence of the fraction of molecules in each state. Specifically, for the model shown in …. (II) the corresponding equations for the fractions of I, X, and U are:

$$f_I = \exp(-k_2 t) \qquad \text{(S1)}$$

$$f_X = [k_2/(k_3 - k_2)]\,[\exp(-k_2 t) - \exp(-k_3 t)] \qquad \text{(S2)}$$

$$f_U = 1 - [1/(k_3 - k_2)]\,[k_3 \exp(-k_2 t) - k_2 \exp(-k_3 t)] \qquad \text{(S3)}$$

At any given time, the value of a physical observable (say, θ) would be

$$\theta = \theta_I f_I + \theta_X f_X + \theta_U f_U \qquad \text{(S4)}$$

where θ_I, θ_X, and θ_U are the characteristic values of the observable for states I, X, and U, respectively. Substitution in S4 of the expressions for each of the fractions given in S2 to S3 leads to

$$\theta = \theta_I \exp(-k_2 t) + \theta_X [k_2/(k_3 - k_2)]\,[\exp(-k_2 t) - \exp(-k_3 t)]$$

$$+ \theta_U \{1 - [1/(k_3 - k_2)]\,[k_3 \exp(-k_2 t) - k_2 \exp(-k_3 t)]\}$$

After factoring of common exponential terms, this latter equation can be written as

$$\theta = \{\theta_I + \theta_X\,[k_2/(k_3 - k_2)] - \theta_U\,[k_3/(k_3 - k_2)]\}\ \exp(-k_2 t)$$

$$+ \{\theta_U\,[k_2/(k_3 - k_2)] - \theta_X\,[k_2/(k_3 - k_2)]\}\ \exp(-k_3 t) + \theta_U \qquad \text{(S5)}$$

Rearrangement of equation (S5) gives

$$\theta - \theta_I = \{(\theta_X - \theta_I)\,[k_2/(k_3 - k_2)] - (\theta_U - \theta_I)\,[k_3/(k_3 - k_2)]\}\ \exp(-k_2 t)$$

$$+ (\theta_U - \theta_X)[k_2/(k_3 - k_2)]\ \exp(-k_3 t) + (\theta_U - \theta_I)$$

By dividing both sides of this last equation by $\theta_U - \theta_I$ (which equals the total observed change), we obtain an expression for the fractional change of the observable:

$$(\theta - \theta_I)/(\theta_U - \theta_I) = \{(\theta_X - \theta_I)/(\theta_U - \theta_I)\ [k_2/(k_3 - k_2)] - k_3/(k_3 - k_2)\}\ \exp(-k_2 t)$$

$$+ (\theta_U - \theta_X)/(\theta_U - \theta_I)\ [k_2/(k_3 - k_2)]\ \exp(-k_3 t) + 1 \qquad \text{(S6)}$$

On the other hand, the expression used for data analysis (see equation 1 in text) would be written as

$$\theta = \theta_0 + A_1[\exp(-\lambda_1 t) - 1] + A_2[\exp(-\lambda_2 t) - 1] + A_3[\exp(-\lambda_3 t) - 1] \qquad \text{(S7)}$$

However, because the first fast phase is effectively decoupled from the other two, only the last two exponential terms were retained for analysis. Taking this under consideration, equation (S7) can be modified to express the fractional change in θ as

$$(\theta - \theta_0)/(\theta_f - \theta_0) = [-A_2/(A_2 + A_3)]\ \exp(-\lambda_2 t) + [-A_3/(A_2 + A_3)]\ \exp(-\lambda_3 t) + 1 \qquad \text{(S8)}$$

where $\theta_f - \theta_0 = -(A_2 + A_3)$ represents the total change in θ associated to the last two exponential terms in (S7). If we recall that for a sequential model $\lambda_2 = k_2$ and $\lambda_3 = k_3$, and recognizing that $\theta_0 = \theta_I$ and $\theta_f = \theta_U$, then a comparison of equations (S6) and (S8) gives

$$-A_2/(A_2 + A_3) = (\theta_X - \theta_I)/(\theta_U - \theta_I)\,[k_2/(k_3 - k_2)] - k_3/(k_3 - k_2)$$

and $$-A_3/(A_2 + A_3) = (\theta_U - \theta_X)/(\theta_U - \theta_I)\,k_2/(k_3 - k_2)$$

From these two equations, the fractional changes of the observable can be calculated from the fractional amplitudes and the rate constants as follows:

$$(\theta_X - \theta_I)/(\theta_U - \theta_I) = k_3/k_2 - [(k_3 - k_2)/k_2]\,[A_2/(A_2 + A_3)] \quad \text{(S9)}$$

$$(\theta_U - \theta_X)/(\theta_U - \theta_I) = -[(k_3 - k_2)/k_2]\,[A_3/(A_2 + A_3)] \quad \text{(S10)}$$
