# Supplementary material for: Complex kinetics and residual structure in the thermal unfolding of yeast triosephosphate isomerase
Source: BMC Biochem. 2015 Sep 3;16:20. doi: 10.1186/s12858-015-0049-2 (PMC4558838; doi:10.1186/s12858-015-0049-2)
Supplement: Additional file 7: — Refolding kinetics of yTIM as followed by CD. (PDF 133 kb) [file 12858_2015_49_MOESM7_ESM.pdf]

Additional file 7

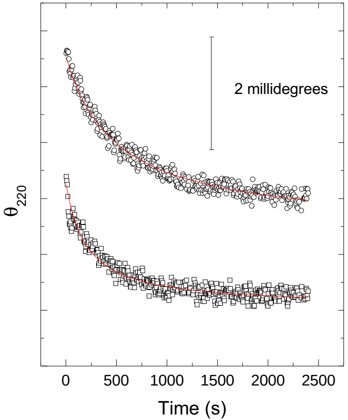

Refolding kinetics of yTIM as followed by far UV-CD. Data shown correspond to pH6.7 (lower trace) and pH8.0 (upper trace). Native yTIM samples (10μg/mL) were allowed to unfold for 10 min at 63.0°C, cooled down to 42.0°C (see Methods) and the ellipticity was then registered. Red lines are least-squares fits of a second-order kinetics equation (see reference 15) to experimental data. Values of the rate constants determined from fit were 6900 (pH 6.7) and 2700 (pH 8.0) $s^{-1}M^{-1}$.
