# Supplementary material for: Complex kinetics and residual structure in the thermal unfolding of yeast triosephosphate isomerase
Source: BMC Biochem. 2015 Sep 3;16:20. doi: 10.1186/s12858-015-0049-2 (PMC4558838; doi:10.1186/s12858-015-0049-2)
Supplement: Additional file 8: — Molecular dynamics simulations of yTIM unfolding. (PDF 113 kb) [file 12858_2015_49_MOESM8_ESM.pdf]

Additional file 8

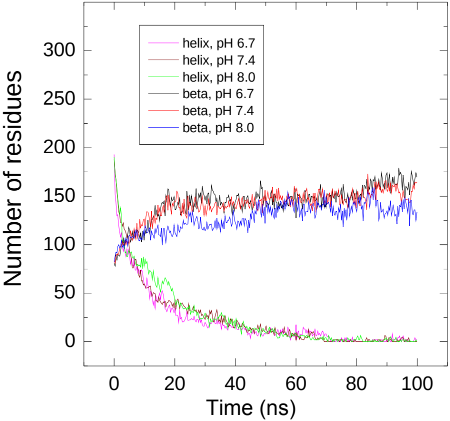

Molecular dynamics simulations of yTIM unfolding at 400K. The evolution of α-helix and β-strand residues is shown.
